# Interaction of tRNA with Eukaryotic Ribosome

**DOI:** 10.3390/ijms16047173

**Published:** 2015-03-30

**Authors:** Dmitri Graifer, Galina Karpova

**Affiliations:** 1 Institute of Chemical Biology and Fundamental Medicine, Siberian Branch of the Russian Academy of Sciences, pr. Lavrentieva, 8, 630090 Novosibirsk, Russia; E-Mail: graifer@niboch.nsc.ru; 2 Department of Natural Sciences, Novosibirsk State University, ul. Pirogova, 2, 630090 Novosibirsk, Russia

**Keywords:** tRNA, eukaryotic ribosome, eukaryotic translation factors, ribosomal RNA, ribosomal proteins, classical and hybrid tRNA states

## Abstract

This paper is a review of currently available data concerning interactions of tRNAs with the eukaryotic ribosome at various stages of translation. These data include the results obtained by means of cryo-electron microscopy and X-ray crystallography applied to various model ribosomal complexes, site-directed cross-linking with the use of tRNA derivatives bearing chemically or photochemically reactive groups in the CCA-terminal fragment and chemical probing of 28S rRNA in the region of the peptidyl transferase center. Similarities and differences in the interactions of tRNAs with prokaryotic and eukaryotic ribosomes are discussed with concomitant consideration of the extent of resemblance between molecular mechanisms of translation in eukaryotes and bacteria.

## 1. Introduction

**Figure 1:**
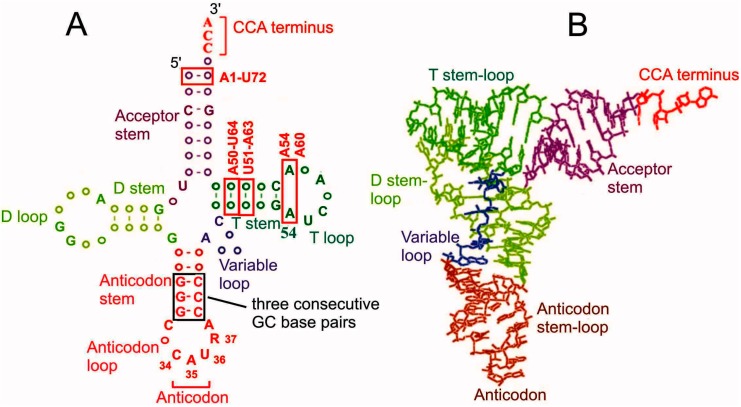
Secondary cloverleaf (**A**) and spatial l-shaped (**B**) structures of tRNA [5]. Structural elements essential for functioning of eukaryotic initiator tRNAs are boxed; elements specific for these tRNAs [2,3,8] are marked as red. Nucleotides conserved in both prokaryotic and eukaryotic initiator tRNAs are given in the cloverleaf tRNA structure (without base modifications) according to the tRNA database [9]. R in position 37 is a hypermodified adenosine.

Amino acid residues necessary for protein synthesis are delivered to ribosomes by tRNA molecules with the aid of special translation factors. Each tRNA is highly specific to a single particular amino acid; tRNA has an anticodon, by which it recognizes its cognate mRNA codon. Correspondence of mRNA codons and amino acid residues is determined by the genetic code, which is almost universal in all domains of life. A number of amino acid (aa) residues can be transported to the ribosome by two or more varieties of specific tRNAs (isoacceptor tRNAs) that recognize different mRNA codons because of the degeneracy of the genetic code allowing coding of one amino acid residue by several mRNA triplets (for review, see [1]). tRNAs are divided into initiator and elongator ones. Initiator tRNAs deliver the first aa residue (always methionine) for protein synthesis and work only at the step of translation initiation while elongator tRNAs carry all other aa residues required in the course of elongation of the nascent polypeptide chain. All tRNAs have a similar cloverleaf secondary and l-shaped tertiary structures. Structural features of tRNAs providing their functioning in the ribosomal translational machinery are well known as well as peculiarities of initiator tRNAs preventing them from working at elongation steps (for review, see [2,3,4]) (Figure 1). In particular, features specific for eukaryotic initiator tRNAs are base pairs A1-U72, A50-U64 and U51-A63, and conserved A54 and A60 in the T loop, whereas three consecutive GC pairs in the anticodon stem are inherent to initiator tRNAs from all domains of life [2,3,4,5,6,7,8].

Mechanisms of translation on ribosomes in bacteria and eukaryotes share significant degrees of similarity, which is most pronounced at the elongation step. Elongator tRNAs are so strongly conserved that bacterial tRNAs can correctly bind to ribosomes from higher eukaryotes displaying the binding properties very similar to those of eukaryotic tRNAs [10,11,12]. However, distinctions in the interactions of tRNAs with bacterial and eukaryotic ribosomes had been also reported. In particular, in an early study [11] it was detected that tRNA binding to the A and P sites of eukaryotic ribosomes in the absence of translation factors is characterized by positive cooperativity, which was never observed with bacterial ribosomes. Later studies provided more details for differences in tRNA binding capabilities of eukaryotic and bacterial ribosomes and their subunits [13] and revealed essential dissimilarities in features of bacterial and mammalian ribosomes related to their modes of interaction with tRNA [14]. These dissimilarities are mainly displayed at the initiation stage of protein synthesis and concern interactions of tRNA with components of ribosomal translational machinery. In eukaryotes initiator methyonyl-tRNA (Met-tRNA_i_) is not formylated and is delivered to the P site of the small ribosomal subunit as a ternary complex with eIF2 and GTP. The mRNA start codon reaches the P site of the small subunit as the result of scanning of mRNA from its capped 5'-terminus by the 43S preinitiation complex containing Met-tRNA_i_, GTP, eIF1, eIF2, eIF3 and eIF1A (homologous to bacterial IF1 (e.g., see [8])). It is worth to note that joining of the large ribosomal subunit to the initiation complex is promoted through the homologous GTPases IF2 and eIF5B, in bacteria and eukaryotes, respectively [8].

Correct functioning of tRNAs during translation is achieved via their proper dynamical positioning within the ribosome mediated by specific interactions with the ribosomal components, the mRNA and the translation factors. Thus, it is obvious that the knowledge on these interactions is of basic importance for molecular biology and can potentially lead to further medical applications. In particular, information on tRNA-ribosome contacts that take place with bacterial ribosomes but do not exist with ribosomes of higher eukaryotes can be used in elaboration of new antibacterial drugs targeted to components of bacterial ribosomes that are involved in contacts of this kind. Besides, knowledge on constituents of the eukaryotic ribosome that are implicated in interactions with tRNA is crucial for understanding molecular mechanisms and diagnosis of genetic diseases related to mutations in genes of the respective ribosomal constituents. Data on positioning of eukaryotic tRNAs on the ribosome and their interactions with components of the translational machinery have been obtained mainly during recent years, by means of cryo-electron microscopy (cryo-EM) [6,15,16,17,18,19,20] and X-ray crystallography [21]. Interactions of acceptor 3'-end of tRNA were also revealed by means of site-directed cross-linking [22,23,24,25] and chemical probing experiments [25]. This review summarizes recently obtained data on interaction of tRNA with translation factors and the cytoplasmic eukaryotic ribosome in classical and hybrid states during initiation and elongation steps.

It is worth to mention here that we have used a new nomenclature for ribosomal proteins (rps) [26]. In this nomenclature letter “u” designates universal (conserved) rps named as the respective bacterial homologs, letter “e” concerns eukaryote-specific or eukaryote/archaea-specific rps and letter “b” designates rps specific to bacteria.

## 2. The Advance of tRNAs through the A, P and E Sites Produces Hybrid Binding States

Ribosomes have three tRNA binding sites, namely, the A (aminoacyl) for incoming aminoacyl-tRNA (aa-tRNA), P (peptidyl) for stable binding of initiator (f) Met-tRNA at initiation and peptidyl-tRNA at elongation of translation, and E (exit) for binding of deacylated tRNA before it leaves the ribosome. Binding of tRNA molecules at these sites in simplified model systems (without translation factors) was studied first with bacterial ribosomes [27,28,29,30,31,32] and then with eukaryotic ones [10,11,12]. In particular, it has been found out that (i) binding of tRNAs to the ribosomal P site depends strongly, and to the A site completely, on the presence of cognate codon and can be observed with either charged or deacylated tRNAs; (ii) binding at the A and P sites is determined mainly by the small ribosomal subunit, which can bind tRNAs independently of the large subunit; (iii) E site binding can take place only with deacylated tRNA, it depends weakly (or does not depend at all) on the nature of codon placed at the E site and occurs mainly at the large subunit; (iv) tRNAs in all forms have higher affinity to the P site than to the A or the E sites. These binding properties were widely used to easily obtain various model complexes of ribosomes with tRNAs for studying positioning of mRNA codons and of tRNA in the ribosome by means of site-directed cross-linking (e.g., [22,23,24,25,33,34]), cryo-EM (e.g., [14,18]) and X-ray crystallography (e.g., [35,36,37,38]) and by other methods, e.g., single-molecule fluorescence resonance energy transfer (FRET) [14,39,40,41].

**Figure 2:**
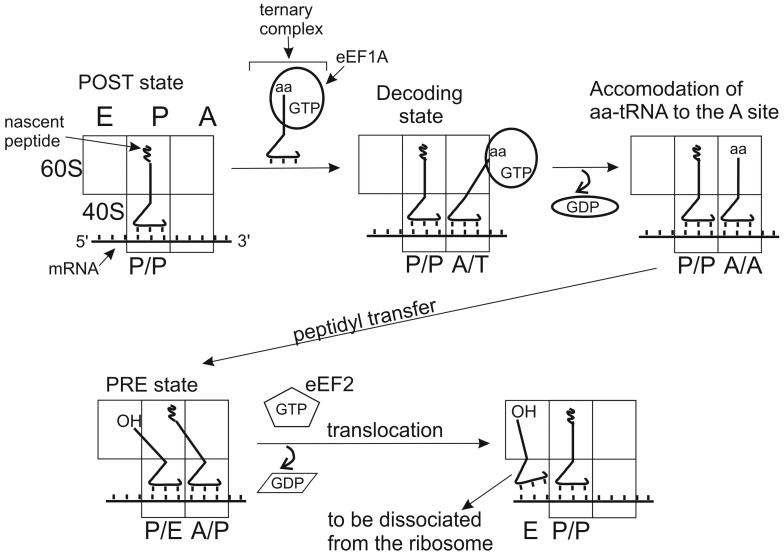
Simplified schematic representation of classical and hybrid states adopted by tRNAs in the course of the elongation cycle on the 80S ribosome. Initially, the P site is occupied with peptidyl tRNA and the A site is free (posttranslocational state, POST). Aminoacyl-tRNA is delivered to the A site within the ternary complex with eEF1A and GTP. If the aa-tRNA is cognate to the mRNA codon bound at the A site, codon-anticodon interaction takes place (decoding). This triggers GTP hydrolysis by eEF1A, which in turn leads to alteration of the factor’s conformation, dissociation of the eEF1•GDP from the ribosome and accommodation of the aa-tRNA to the A site. As the result, the acceptor end of the aa-tRNA becomes free and appears at the peptidyl transferase center, allowing fast transfer of the nascent peptide chain to the A site bound aa-tRNA (transpeptidation). After this, the acceptor end of the A site tRNA spontaneously moves to the P site (hybrid A/P state) and the acceptor end of the deacylated P site tRNA to the E site (P/E state); the ribosomal complex formed corresponds to the pretranslocational (PRE) state. Binding of ribosomal GTPase eEF2 to the PRE complex promotes translocation of the tRNAs with the bound mRNA codons, which results in formation of the new POST state, where deacylated tRNA is bound at the E site before it leaves the ribosome and the A site is ready to accept aa-tRNA cognate to the next mRNA codon.

Positioning of tRNA molecules at the A and P sites depends on the functional state of the ribosome, which has been first demonstrated with pre- and postranslocational (PRE and POST) complexes of *E. coli* ribosomes [42,43]. PRE is the state preceding ribosomal translocation with peptidyl-tRNA at the A site and deacylated tRNA at the P site. POST is the state after translocation when peptidyl-tRNA occupies the P site and deacylayted tRNA is at the E site. Studying protections of rRNA nucleotides from chemical modification by ribosome-bound tRNAs lead to a conclusion that tRNAs at the A and P sites prior to translocation adopt hybrid (intermediate) states (A/P and P/E). In these states, anticodon domain of tRNA interacts with the mRNA codon in one site (A or P) at the small subunit, while the acceptor domain interacts in the large subunit with a region corresponding to the site, to which it is going to translocate (P or E, respectively) (Figure 2).

In addition to A/P and P/E states, hybrid P/I and A/T states of tRNA are now well recognized [44,45,46,47]. P/I is the state of Met-tRNAi in the preinitiation complexes (PICs) where the CCA-terminus is lifted out of the position that it occupies when bound at the peptidyl transferase center (PTC) of the assembled 80S ribosome (e.g., see [45,46,47]). A/T is the state, in which aa-tRNA is bound at the ribosomal A site within the ternary complex with elongation factor EF-Tu (bacteria) or eEF1A (eukaryotes) and GTP. The CCA terminus of tRNA in this state interacts mainly with the factor and is away from the PTC at the large subunit. The acceptor terminus of aa-tRNA can reach the PTC only after ribosome-induced GTP hydrolysis, which transfers aa-tRNA from A/T to the classical A/A state (e.g., see [44] and refs therein).

Classical and intermediate hybrid tRNA states have been visualized in numerous cryo-EM studies with bacterial [48,49] and recently with eukaryotic [14,18,20] ribosomal complexes. These studies showed that hybrid states formation is coupled to alterations of mutual orientation of ribosomal subunits. These alterations include ratchet-like rearrangement (which is induced by EF-G/eEF2 binding) and a swivel movement of the small subunit head that take place in both prokaryotic and eukaryotic ribosomes, and subunits rolling specific to eukaryotic ribosomes (will be discussed below).

## 3. Networks of tRNA Interactions Change in the Course of Its Pass through the Stages of Translation Initiation

During translation initiation in eukaryotes, Met-tRNA_i_ interacts with the small ribosomal subunit, start codon of mRNA and several initiation factors including eIF2 and eIF5B. These interactions are discussed in detail below.

### 3.1. Interactions of Met-tRNA_i_ with eIF2

In bacteria, fMet-tRNA_i_ binds directly to the P-site of the small ribosomal subunit containing AUG codon of the mRNA, and IF3 controls the fidelity of this process. In eukaryotes, Met-tRNAi is selected by a designated factor eIF2 (composed of three subunits α, β and γ) and is delivered to the eukaryotic 40S ribosomal subunit at an early step of translation initiation within its specific ternary complex (TC) with eIF2 and GTP. The eIF2 has a homologue in archaea (aIF2) but not in bacteria (e.g., for review see [50]). Molecular details of interactions of Met-tRNA_i_ with the factor are better known with the archaeal TC whose structure has been solved by X-ray crystallography [51,52]. According to the 5 Å resolution structure obtained in [51] that contains all three subunits of aIF2 (in contrast to that reported in [52], where the β subunit was lacking), the tRNA is bound to the α and γ subunits of aIF2, and the contacts involve the elbow of the tRNA and the minor groove of the acceptor stem. The authors note that, despite considerable structural homology between the core γ subunit of aIF2 and the elongation factor EF1A, these two translational GTPases use very different tRNA-binding strategies, which can be rationalized because functions of these factors imply that they should not confuse initiator and elongator tRNAs. Various data were obtained on contributions of subunits of aIF2 and eIF2 to affinities of these factors to the tRNA. In archaea, the α subunit provides the main contribution to the aIF2 affinity to tRNA; the isolated γ subunit can also bind tRNA but its affinity to tRNA is much weaker than that of the heterotrimer, and the contribution of the β subunit in tRNA binding is small [51,53,54,55]. On the contrary, in eukaryotes the α subunit only slightly contributes to the affinity of eIF2 for tRNA binding, while the β subunit plays an important role in the binding (this was dubbed an “eukaryotic behavior” [56]). These data obtained by biochemical approaches without ribosomes disagree with the results of a cryo-EM study of the mammalian 43S PIC (solved at 11.6 Å resolution), in which the TC was bound to the 40S subunit [16]. In this complex strong interaction between eIF2α (namely, its domain 2, D2) and the tRNA was observed while no contacts with eIF2β were detected. This discrepancy could arise from large rearrangements of the eIF2α conformation accompanying binding of the TC to the 40S subunit observed in the mentioned study. These rearrangements enable eIF2α to interact with rp uS7, which has been independently demonstrated in [57] with 48S PICs assembled in rabbit reticulocyte lysate with different model mRNAs. The discussed reorientation of eIF2α was observed as well in the recently reported cryo-EM structure of yeast 48S PIC at 4 Å resolution [58]. It is worth to note here that there are two different states of Met-tRNA_i_ in the PICs, one exists in the 43S complex before mRNA binding to the small subunit and another takes place in the 48S complex containing mRNA. This calls forth differences in interactions of tRNA_i_ with eIF2 in these complexes (for detail, see the next section).

### 3.2. Interactions of Met-tRNA_i_ in PICs with the 40S Subunit

It is generally accepted that in PICs there are two modes of TC binding, P_out_ and P_in_ (e.g., see [8,59]). P_out_ takes place in 43S PICs formed before mRNA binding and containing TC, eIF1 and eIF1A as well as in 48S PICs, in which the 40S subunit is scanning mRNA but has not yet reached the initiation AUG codon. In this case the PIC is in open (scanning competent) conformation and tRNA is not fully bound at the P site. When the start AUG codon becomes recognized at the P site, *i.e.*, base paired with the Met-tRNA_i_ anticodon, the tRNA completely occupies the P site. This state corresponds to scanning incompetent closed conformation of the PIC and P_in_ mode of tRNA binding. It is suggested that transitions between P_out_ and P_in_ modes are mediated by direct interactions of tRNA with regulatory sequences in factors eIF1 and eIF1A. In particular, the interactions with scanning enhancer elements in eIF1 and in the C-tail of eIF1A stabilize the open conformation of the PIC and the “P_out_” mode of tRNA binding. On the contrary, interactions with scanning inhibitor elements in the *N*-terminal and helical domains of eIF1A stabilize the closed conformation of the PIC and P_in_ mode of tRNA binding [8,59].

Positioning of Met-tRNA_i_ in the mammalian 43S PIC assembled without mRNA was studied by cryo-EM at 11.6 Å resolution [16]. In this complex the anticodon of Met-tRNA_i_^Met^ is positioned just in the P site and the elbow is tilted toward the E site as compared to the classical P/P orientation; this tilt is similar to those in P/I and P/I1 orientations of initiator tRNAs in prokaryotic initiation complexes [45,60,61]. For all of that, orientation of the tRNA in the 43S PIC was closer to the P/I state (in which the acceptor end is shifted in the direction of the E site [45,60]) than to the P/I1 state (where the CCA end is oriented toward the A site [61]). A feature peculiar to the eukaryotic complex is that the tRNA elbow is shifted in the direction of the head of the 40S subunit, and the authors termed this tRNA state as eukaryotic P/I or eP/I [16]. Comparison of these data with later cryo-EM structure of the yeast 48S complex assembled with mRNA [58] showed differences in tRNA positions in the 43S and 48S complexes, in particular, in the latter tRNA was positioned deeper in the P site than in the 43S complex. The mentioned differences are compatible with those between a P_out_ state of tRNA_i_ expected with the 43S complex and a P_in_ state expected with the 48S complex. It was found that Met-tRNA_i_ in the 48S complex adopts a eP/I conformation [58] similar to that observed in the 43S complex [16].

**Figure 3:**
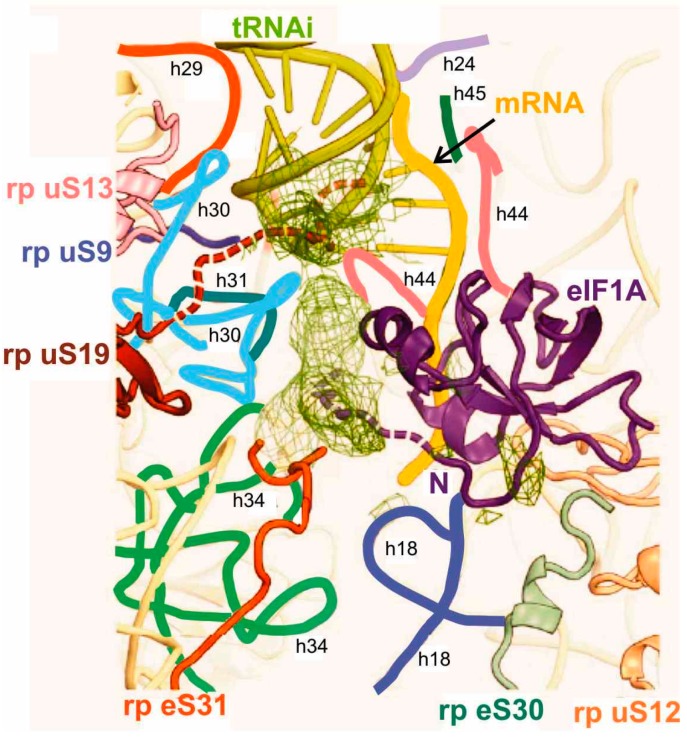
Binding pocket for the codon-anticodon duplex at the P site of the model mammalian 48S PIC [21]. Proposed locations of the *C*-terminus of rp uS19 (brown) and *N*-terminal tail of eIF1A (violet) are shown as dashed lines. Helices (h) of the 18S rRNA are marked with individual colors and designated with small letters.

Structural information on the tRNA interactions in the mammalian PICs was obtained by X-ray crystallography at a resolution limit of 7 and 8–9 Å depending on the complex type [21]. In the 48S complex, the binding region for the anticodon stem-loop (ASL) of Met-tRNA_i_^Met^ comprised 18S rRNA helices h28 and h44 of the body, h24 of the platform and h28, h29, h30 and h31 of the head of the 40S subunit together with ribosomal proteins (rps) uS19, uS16 and uS13 located at the subunit’s head (see Table 1 and Figure 3).

**Table 1:** Several contacts of tRNA in eukaryotic ribosomal translational complexes. Eukaryote-specific interactions are shown in bold.

tRNA Fragment	Binding Partner			
	P Site		E Site	A Site
	Initiation	Elongation		
Anticodon loop	rp uS9 (C-tail) and 18S rRNA h30, h31 and h44 (top) (48S complex [21])	rp uS9 (C-tail) and 18S rRNA h30, h31 and h44 (top) [14,15,18,20]	18S rRNA h28, h29, loop h29-h42 and rp uS11 [14]	18S rRNA 530 loop, h34 and h44 (top) [14]
Anticodon stem	18S rRNA h24, h28 and h29 (48S complex [21])	18S rRNA h24a, h30, h43 and h44 (top) [14,15]	18S rRNA h23b, loop 690 [14] and rp uS5 [14,18,20]	18S rRNA h18, h30, 965 loop, rp uS19, rp uS12 and/or rp eS30 [14]
D stem-loop	***N*****-terminal domain 2 (D2) of eIF2α** (43S complex [16])	28S rRNA H69 [15,18,20] and **rp eL44 (C-tail)** [18,20]	28S rRNA loop H77-H78 ** [14]	28S rRNA H69, H38 (“A site finger”) [14], **rp uS12 and H89 in A/T state** [20]
T stem-loop		rp uL5 * [15,18,20], **eL44 (C-tail)** [18,20] and 28S rRNA H85, loop H82-H83 * [14]	rp uL1 ** [14,18,20] and 28S rRNA loop H77-H78 ** [14]	28S rRNA H89 [14], **rp uS12 and sarcin-ricin loop (apical loop of 28S rRNA H95) in A/T state** [20]
Acceptor stem	*C*-terminal domain of eIF5B [6,19]	28S rRNA H80 * [14,15] and H93 [15]	28S rRNA H68 ** [14]	28S rRNA loop H69-H71 [14]
CCA-end	28S rRNA H80 [6], and **domain IV of eIF5B** [6,19] (80S complex)	28S rRNA H93, the PTC ring [14,25] and H89 [25]	28S rRNA H82 [18], loop H74-H88 ** [25] and **rp eL44** ** (region of K53 in human numbering) [18,20,23,24]	28S rRNA H89 [14,25], H92, and H93 [25]
5'-end	not analyzed	**Rp uL16** [15,20]	not analyzed	not analyzed

* Contacts taking place also in A/P state [14]; ** Contacts taking place also in P/E state [18,20].

**Figure 4:**
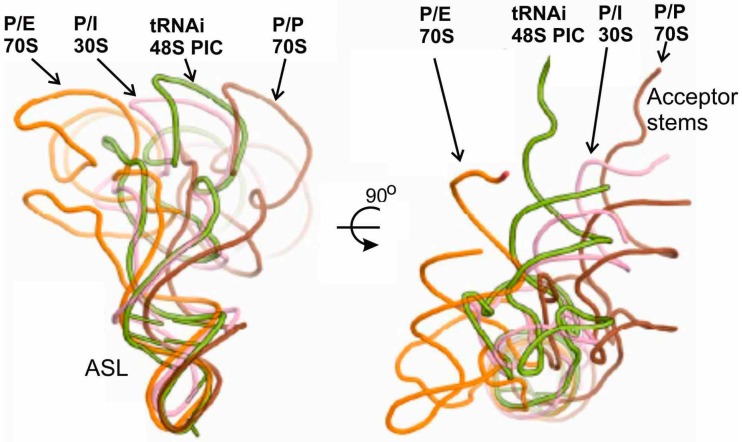
Comparison of positioning of tRNA in the P/I state in the 48S PIC with that in the bacterial 30S IC and with those in P/E and P/P states in the 70S complexes [21].

Interactions of tRNA_i_ with rRNA were similar to those seen for the P site tRNA bound to the 70S ribosome [37,62]. It is worth to note that the rRNA was arranged differently in the 48S PIC and in 40S complexes containing eIF1 and eIF1A but free of mRNA and tRNA [21]. The authors attributed these dissimilarities to the differences between the scanning-incompetent and scanning-competent conformations that bind tRNA_i_ in the P_in_ and P_out_ modes, respectively [21]. The crystallographic data have also shown that transition from P_out_ to P_in_ states is accompanied by rotation of the head of the 40S subunit, which locks tRNA_i_ in the P_in_ state and completes the scanning. Besides, the structures suggested that the tRNA binding in the P_in_ mode in the 48S PIC could be stabilized by both the *N*-terminal tail of eIF1A as mentioned above [8,59] and the *C*-terminus of rp uS19 (Figure 3). In addition, the X-ray models have clarified two structural points essential for understanding molecular mechanisms of the initiation process [21]. First, it was found that eIF1A interacts with the *N* terminus of rp eS30 and with rp uS12 (Figure 3), which makes the A site inaccessible to elongator tRNAs during initiation. Second, it was shown that eIF1 spatially interferes with the adjustment of the ASL of the initiator tRNA in the P site during scanning. Upon the correct codon–anticodon interaction, eIF1 cannot compete with the ASL for the P site and must dissociate, which occurs when the 48S PIC is formed. Finally, the discussed X-ray study [21] illustrates the extent of similarity and distinctions between various states of tRNA at the P site of bacterial and eukaryotic ribosomes (Figure 4). Observed differences in the conformation of factor-free bound tRNA in the 70S complexes and of tRNAi bound in the 48S PIC and the 30S IC allowed authors to suggest that eIF5B (a GTPase homologous to bacterial IF2) might orient the acceptor stem of tRNAi toward the P site on the approaching 60S subunit to promote its joining to the 48S PIC. This hypothesis was later elaborated and confirmed by cryo-EM study of the mammalian 80S initiation complex [19].

### 3.3. Interactions of Met-tRNA_i_ in 80S Initiation Complexes

The 80S initiation complex is formed at the last step of translation initiation when joining of the 60S subunit and the 48S PIC occurs. After GTP hydrolysis in the ribosomal complex, initiation factors dissociate making the complex competent for elongation. Interactions of Met-tRNA_i_^Met^ were studied by cryo-EM in 80S initiation complexes containing eIF5B and a non-hydrolysable GTP analogue. These complexes were assembled in mammalian system with IRES element of hepatitis C virus RNA (HCV IRES) [19] and in the yeast system with mRNA lacking IRES [6] and solved at resolutions about 9 and 6.6 Å, respectively.

In the complex with mRNA lacking IRES, the CCA-end of the tRNA was found in contact with the β barrel of the eIF5B domain IV in the vicinity of the PTC (Table 1), preventing the CCA end to reach the PTC and the formation of an elongation-competent complex until GTP hydrolysis and dissociation of eIF5B from the ribosome take place. Contact of eIF5B with the 3' end of tRNA in the ribosome was enabled due to large conformational change in the factor upon ribosome binding, and it was suggested that this change couples initiator tRNA recognition to GTP hydrolysis. Besides, it was found that in the 80S complex, ASL of the Met-tRNA_i_^Met^ is slightly displaced from the position corresponding to that of P-site tRNA in the 70S ribosome, , which is typical for the P/I state of tRNA observed earlier in the respective bacterial 70S complexes [60]. It is worth to note that in the 80S complex the subunits are rotated relative to the orientation characteristic for ribosomal complex after initiation [6]. A finding that *C*-terminal domain of eIF5B makes extensive contacts with A1-U72 pair (Table 1) was proposed to indicate specific recognition of this pair in the initiator tRNA by the factor [6]. The bent of the Met-tRNA_i_^Met^ at the anticodon stem exactly in the region characterized by three consecutive GC pairs peculiar to all initiator tRNAs was suggested to allow the bent conformation to be stabilized by eIF5B, enabling the tRNA to place the CCA-end out of the PTC [6].

**Figure 5:**
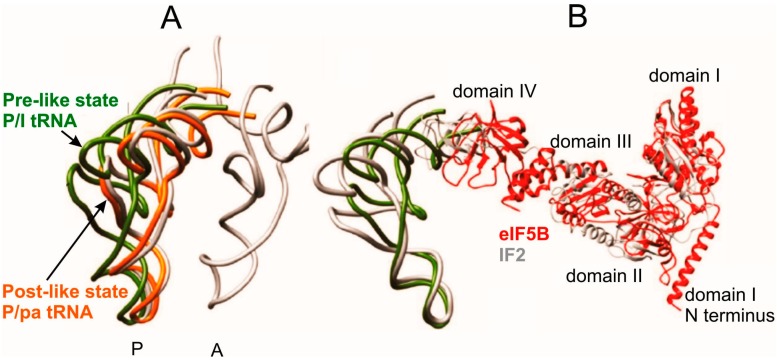
Comparison of tRNA positioning in the Pre-like and Post-like states of the 80S initiation complex with that in classical A and P sites [19]. (**A**) configurations of P/I-tRNA in the Pre-like state (green), P/pa-tRNA in the Post-like-state (orange) and classical A/A-tRNA and P/P-tRNA (gray); (**B**) comparison of the positioning of Met-tRNA_i_^Met^ and eIF5B (red) within the mammalian Pre-like state and that of IF2 and fMet-tRNA_f_^Met^ within the bacterial initiation complex (gray).

In the study on the mammalian 80S initiation complexes assembled with HCV IRES, two subpopulations of the complex were observed, which differed from each other by the rotational state of the ribosomal subunits and positioning of Met-tRNA_i_^Met^ in the P site [19]. The structure of one subpopulation was in general similar to that described in [6] and was named “Pre-like” with tRNA in the P/I state. Formation of the Pre-like 80S initiation complex promoted by eIF5B is accompanied by rolling of the 40S subunit, a rotation of the small subunit around its long axis that has no analogy in bacterial ribosomes. Another subpopulation of the IRES-containing 80S complex represented unseen previously conformation, which was a result of the subunit reverse rolling from the Pre-like to the Post-like ribosomal subunit configuration. In the “Post-like” complex, tRNA was present in a unique state where the ASL and the elbow were at P site, and the 3'-CCA end at the A site; this tRNA state was denoted as the P/pa state [19] (Figure 5). The authors consider the Pre-like state as a state occurring immediately after subunit joining, and the Post-like state as an intermediate state during the transition of the Pre-like state to the elongating POST one. In the course of initiation, the CCA-terminus of Met-tRNA_i_^Met^ is expected to shift from the P/pa configuration (Post-like state) into the canonical P/P position upon release of eIF5B from the ribosome. In the “Pre-like” state tRNA acceptor stem interacts mainly with domain IV of eIF5B and barely contacts the ribosome (Table 1). This interaction was suggested to facilitate movement of domain IV of eIF5B relative to the 60S subunit during rolling of the 40S subunit to guide Met-tRNA_i_^Met^ bound to the domain IV from the P/I to the P/pa state [19]. It was assumed that P/pa state of tRNA can be realized for HCV-IRES-driven internal initiation as well as for canonical translation initiation.

## 4. Dynamics of tRNA Interactions during Translation Elongation

In the course of elongation, two essential ribosomal states are generally considered, PRE and POST ones; both types of 80S ribosomal complexes were studied in detail, mainly by cryo-EM.

### 4.1. Interactions in POST Complexes

The first study on structural aspects of interactions of tRNA with the eukaryotic ribosome was a cryo-EM study with yeast 80S ribosome containing peptidyl-tRNA at the P site [15] (POST state with classical P/P configuration of the tRNA) solved at 15.4 Å resolution. Suggestions on tRNA-ribosome interactions based on this low-resolution structure were in general confirmed in the recent more detailed study of yeast POST-like complex containing tRNA molecules at the P and E sites of the nonrotated 80S ribosome solved at 6 Å resolution [18] (tRNA-ribosome contacts deduced from this study are summarized in Table 1). Conformation of tRNA bound at the P site of the POST 80S ribosomal complex is somewhat different from that of the free tRNA and those observed earlier with bacterial ribosomal complexes [35,63]. Nevertheless, the binding pocket for the tRNA resembles that in the respective bacterial complexes. In general, interactions of P/P tRNA with the 80S ribosome are as following. The ASL is bound to the 40S subunit between the head and the body/platform side contacting the apical loop of the 18S rRNA helix h24 in the platform, and from the opposite side, tRNA interacts with 40S components belonging to the head (h30, h31, h43 and the C-tail of rp uS9) (Table 1). The binding pocket for the ASL is very similar to that described above for tRNA_i_ bound in the 48S PIC (Figure 3). The remaining three tRNA domains are bound to the 60S subunit, in particular, the D and T loops contact H69 of the 25S rRNA and rp uL5, respectively, the acceptor stem interacts with H80 (5'-side) and H93 (3'-side), and 5'-terminus with rp uL16 (Table 1).

**Figure 6:**
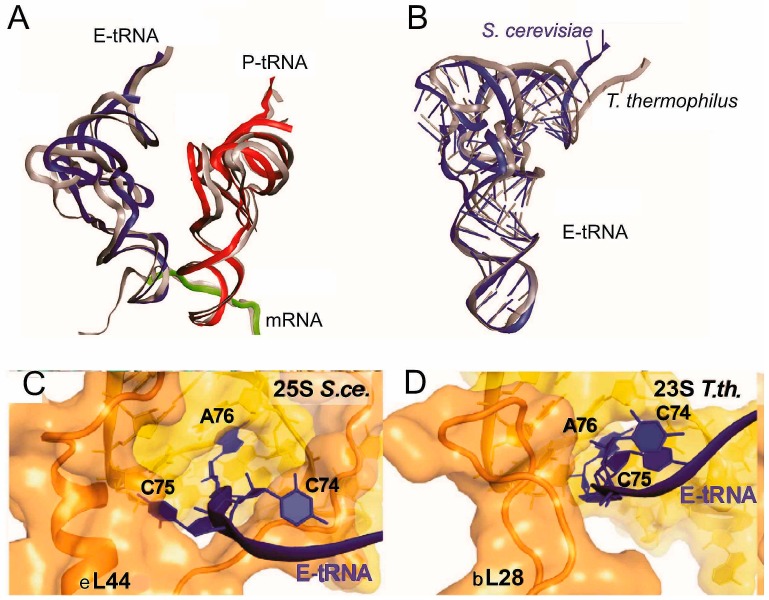
Structural differences between positioning of tRNA in yeast and bacterial POST-like complexes [18]. (**A**) conformational and positional differences between tRNAs bound to yeast (red and dark blue) and bacterial *T. thermophilus* ribosomes (gray); (**B**) conformational difference between E-site tRNAs bound to yeast (dark blue) and bacterial (gray) ribosomes; (**C**,**D**), differences between arrangements of the CCA-termini of the E site tRNAs bound to the yeast (*S.ce.*) and Thermus thermophilus (*T.th.*) ribosomes.

With yeast POST 80S complexes, two global distinctions from the analogous 70S complexes [38] have been revealed [18]. First, the small subunit was significantly tilted relative to the large 60S subunit and the tilt was coupled with displacement of the intersubunit bridge B1b between the head of the 40S subunit and the central protuberance (CP) of the 60S subunit. The second distinction concerned the positions of tRNAs in the classical P and E sites. In particular, in the 80S complex, the P site tRNA was located closer to the E site, whereas the E-site tRNA was closer to the P site, which brought elbows of the tRNAs 15 Å closer than in the respective 70S complexes. The positioning of the P site tRNA in the 80S complex was changed because of altered placement of the CP, which interacts with tRNA via uL5. Altered positioning of the E site tRNA was due to the absence of the contact between the L1 stalk and the small subunit existing in the 70S complex. The most pronounced differences concerned positioning of the CCA-terminus of E site tRNAs in 70S and 80S complexes (Figure 6). In particular, in the bacterial complex the CCA end of the tRNA contacts H82 of 23S rRNA and rp bL28 [37,64], and the CCA end cytosines are stacked with nucleotides of the acceptor stem. In contrast, in the yeast complex the penultimate nucleotide C75 of the E site tRNA is unstacked from C74 and interacts with eukaryote/archaea-specific rp eL44 (Table 1). Only interactions of the CCA with H82 are common in the bacterial and yeast complexes [18]. Interactions of the CCA-terminus of the E site tRNA discussed here turned out to be very similar to those observed earlier in the archaeal complex [65]. In general, similar data were obtained with the mammalian POST complex in a study [20] aimed mainly on the investigation of PRE complexes (discussed in detail below). It is worth to note here that none of the papers discussed above provided a reasonable hypothesis concerning functional assignment of such large differences in the interactions of the CCA-end of the E site tRNA with the ribosome in bacteria and archaea/eukaryotes.

### 4.2. Interactions in PRE Complexes

The first study with PRE 80S complexes was carried out on a mammalian system exploiting cryo-EM at 9–10.6 Å resolution together with single-molecule FRET [14]. The ribosomal complex containing eEF1, aa-tRNA and GMPPNP was observed in two subpopulations in a nonrotated conformation containing classically configured tRNAs in the A, P, and E sites. Two other subpopulations were observed in a rotated conformation with tRNAs in hybrid states. In the two “classical” subpopulations positioning of tRNA at the A site was nearly identical to that in the bacterial 70S PRE complex [35,37,63,65,66], although some of tRNA-ribosome interactions (Table 1) were not observed in the bacterial complex [14]. By contrast, configurations of tRNA in the P/P and E/E states differed substantially from those in the bacterial complexes, from each other and from those in the A/A state. The main differences concerned the elbow region, indicating that classical tRNA configurations are specific in each domain of life.

It was demonstrated that in the two “hybrid” 80S complex subpopulations the P site tRNA was in the same P/E state, while the A site tRNA was in different configurations. In one subpopulation it adopted a classical-like (A/A) configuration. In another one it was in a hybrid (A/P) state, in which orientation of its ASL was similar to that of tRNA in the A/A configuration, but the acceptor stem and the elbow were displaced toward the P site where the elbow contacted the typical P site components such as the central protuberance and rp uL5 (Table 1). PRE complex with (A/A + P/E) states of tRNAs was never observed with the bacterial PRE complexes [67,68]. Analysis of the results has led the authors of [14] to propose that movements of the A-site tRNA elbow toward the P site (to form the A/P configuration) require flexion/bending of the tRNA body. This in turn suggested that conformational changes within the tRNA contribute to the hybrid state formation and tRNA plays an active role in the mechanisms of protein synthesis. Besides, a conclusion has been made that 80S ribosomes prefer the rotated state to nonrotated and stronger prefer hybrid tRNA configurations to classical as compared to 70S ribosomes [14].

The results of the mentioned study turned out to be in agreement with data on interactions of the CCA-terminus of the A site tRNA with the 28S rRNA in PRE-like mammalian 80S ribosomal complexes obtained by means of biochemical approaches. These approaches included site-directed cross-linking with application of tRNA analogues bearing 4-thiouridines at the acceptor end [22,25] and hydroxyl radical probing of the 28S rRNA structure in the region of the PTC in complexes with both full size tRNA and its truncated form deprived of the CCA-end [25] (Table 1). Cross-linking showed that contacts of CCA-end of the A site tRNA are located in regions corresponding to both the A site and the P site positions of the tRNA acceptor ends in the archaeal 50S subunit, indicating that A site tRNA was present at both A/A and A/P states. However, the data of hydroxyl radical probing revealed only the A/P state, which was attributed to an unstable nature of the 80S ribosomal A/A state [25]. It was concluded that this state could be trapped only by cross-linking [25], which was in agreement with the above-mentioned conclusion that 80S ribosomes prefer hybrid tRNA configurations to classical ones [14]. Data obtained by biochemical approaches provided exact identification of 28S rRNA nucleotides interacting with the CCA end of the A site tRNA, which was impossible in the mentioned cryo-EM study [14] because of relatively low resolution (about 10 Å) insufficient for direct visualization of the CCA end.

The more recent cryo-EM study at 7–9 Å resolution (depending on the complex type) provided a number of new details of tRNA-ribosome interactions in the PRE state as well as in intermediate tRNA states preceding the PRE complex formation [20]. In particular, two subpopulations of the 80S ribosomal decoding complex containing the ternary complex Val-tRNA•eEF1A•GMPPNP at the A site and a peptidyl-tRNA analogue at the P site were revealed that were assigned to particular steps of recognition of the cognate aa-tRNA bound in the A/T state. This state precedes the classical A/A state that is realized as the result of accommodation of the aa-tRNA to the A site. One sub-state of the A/T tRNA represented the earliest step of the recognition referred to as initial codon sampling state. In this state codon-anticodon interaction occurred but was not yet stabilized because bases of A1824 and A1825 (A1492 and A1493 in *E. coli* 16S rRNA numbering) were not flipped out to allow minor groove interactions with the codon-anticodon helix to be realized. The GTPase of eEF1A in this sub-state was not activated because the factor did not interact with the sarcin-ricin loop (SRL) of the 28S rRNA (Table 1). Interestingly, the initial codon sampling state was not experimentally observed with bacterial ribosomes.

Another subpopulation of the decoding complex represented the next step of the tRNA recognition, the codon-recognition/GTPase activation state. In this sub-state, the G domain of eEF1A interacted with SRL of the 28S rRNA and bases of A1824 and A1825 were flipped out as well as in the PRE complexes where aa-tRNA was accommodated to the A site (Table 1). Stabilization of the codon-anticodon duplex by A-minor interactions with A1824/A1825 of 18S rRNA and long-range signaling of this occurence to the GTPase center of eEF1A are key events for decoding that are similar in bacterial and eukaryotic complexes. The codon-recognition/GTPase activation state resembled that in the corresponding bacterial 70S complex carrying EF-Tu, aa-tRNA and a non-hydrolysable GTP analogue [69]. With 80S complexes, it was observed that transition from the initial sampling state to the codon recognition/GTPase activation state is accompanied with rearrangements resembling “domain closure” in the 30S subunit [70], which has been described for the first time as a conformational rearrangement of the 30S subunit induced by binding of cognate tRNA at the A site. It includes shoulder movement and head rotation resulting in tightening of the ribosomal structure around the ASL of the A site tRNA [70]. Comparison of the ribosomal structures of the initial sampling state and the codon recognition/GTPase activation state in the 80S ribosome showed that “domain closure” became completed only in the classical PRE states [20]. A specific feature of this rearrangement in eukaryotes was that ASL of aa-tRNA became more deeply placed in the decoding cleft as compared to that in the corresponding bacterial complexes [20]. Besides, accommodation of aa-tRNA to the A site, *i.e.*, the transition from the A/T to the classical A/A state, was accompanied with the subunit rolling mentioned above when the formation of the Pre-like 80S initiation complex promoted by eIF5B was discussed (see Section 3.3). Rearrangement of this kind does not occur during elongation in the bacterial system [69,71,72]. Back subunit rolling was shown to accompany translocation, *i.e.*, conversion of the PRE to the POST state where the ribosomes are in the nonrotated, nonrolled conformation, similar to that in the decoding complexes in the initial codon sampling state [20].

Positioning of the A/T tRNA molecule in bacterial and eukaryotic decoding complexes is not identical. Dissimilarities concern mainly the tRNA elbow, the changed position of which in the 80S complex is stabilized by specific interactions with the 60S subunit [20]. In the mammalian initial codon sampling state, the T- and D-loops of tRNA make the contacts with the SRL and 28S rRNA helix H89, respectively (Table 1), which was suggested to stabilize binding of the A/T tRNA. Comparing the structures of bacterial and mammalian decoding complexes, the authors of [20] revealed the conserved and divergent features of the mammalian complex. Conserved ones are the interactions of the shoulder region of the 40S subunit with two evolutionary conserved loops of domain II of eEF1A and the CCA-end of the aa-tRNA. Divergent features relate to the eEF1A location that is somewhat changed due to the appearance of an eukaryote-specific helical insertion, and to the elbow position of tRNA enabling its interaction with the apical loop of the highly conserved SRL and helix H89 (Table 1). This interaction of the A/T tRNA elbow is specific to the eukaryotic complex and indicates a more rigid and tighter binding of the tRNA elbow to the ribosome.

The 70S and 80S ribosomal PRE complexes present conformational heterogeneity characterized by spontaneous intersubunit rotation and fluctuations between classical and hybrid tRNA configurations (for review, see [73]). In contrast, the POST state of the 80S ribosomal complex was observed in a single conformation with two tRNAs in classical P and E sites, which was assigned to a deep minimum of energy/enthalpy in the energy landscape of the elongating ribosome [20].

It is worth noting that positioning of the P and E site tRNAs in mammalian PRE complex [14] is similar to positioning of those in the yeast POST-like complex [18]. This finding has led the authors of [18] to a conclusion that these tRNA positions are conserved in eukaryotes and that the occupancy of the A site does not affect ribosomal conformation and configuration of tRNAs at the P and E sites.

### 4.3. Interactions in Model 80S Complexes with a Single Deacylated tRNA at the P Site

Stable complexes of ribosomes containing a single molecule of deacylated tRNA at the P site occupied with mRNA codon cognate to this tRNA can be easily obtained without translation factors. These simplified model complexes have been widely used in various studies of both prokaryotic and eukaryotic ribosomes (e.g., for review see [74]). Interactions of the acceptor terminus of deacylated tRNA bound to the P site of human ribosomes were studied by site-directed cross-linking with application of tRNA derivatives containing either terminal ribose oxidized to 2',3'-dialdehyde [23,24] or a 4-thiouridine in the acceptor end [22,25] as well as by hydroxyl radical probing (a footprinting approach) mentioned above [25]. These studies gave the first experimental data on intimate contacts of the acceptor end of the P site tRNA in the 80S ribosome and provided indications for different dynamic properties of ribosomes of prokaryotes and eukaryotes. It turned out that cytidines of the CCA-end of the P site tRNA, especially C75, contact K53 of eL44 (L36AL in human) [24] (Table 1). Since this interaction is typical for the E site (see above), it was concluded that the cross-link of the deacylated tRNA to rp eL44 was from the hybrid P/E state [24]. By analogy, the tRNA analogue with 3'-terminal 4-thiouridine instead of A cross-linked to a 28S rRNA nucleotide in a loop between H74 and H88 (Table 1) located close to rp eL44 in the E site region [25]. Remarkably, K53 of eL44 lies close to the 49-GGQ-51 tripeptide conserved in rps of the eL44 family and identical to the universally conserved motif of class 1 release factors implicated in triggering peptidyl-tRNA hydrolysis (see [24] and references therein). One could assume that contact of cytidines of the CCA-end of the P site tRNA with this amino acid residue is required to ensure transfer of the peptidyl moiety to the aa-tRNA at the A site through the involvement of the eL44 tripeptide 49-GGQ-51 in hydrolysis of the P site peptidyl-tRNA. This suggestion agrees with cryo-EM data revealed P/E state of peptidyl-tRNA and A/P state of aa-tRNA in PRE ribosomal complex formed in the presence of non-hydrolyzable GTP analogue [20] (see Section 4.2).

Notably, a cross-linking approach utilizing 4-thioU-containing tRNA analogues allowed detection of not only P/E but P/P state of tRNA as well [25]. Interestingly, the P/P state was not detected by the footprinting approach. This finding was attributed to what might be the unstable nature of the P/P state of the 80S complex in analogy to the suggestion concerning the A/A state [25] mentioned above.

Finally, it is worth to note that site-directed cross-linking with application of photoactivatable mRNA analogues and their DNA copies (the same oligomers lacking 2'-OH groups) made it possible to reveal the crucial eukaryote-specific role of the 2' hydroxyl group of codon ribose for the tRNA-dependent binding at the P site [75]. This finding indicated differences in arrangements of codon-anticodon duplexes at the P site of bacterial and eukaryotic ribosomes. Being applied to the available atomic structures of lower eukaryotic ribosomes, these data allowed estimation of possible eukaryote-specific contact of the phosphate between 18S rRNA nucleotides 1760 and 1761 (*Saccharomyces cerevisiae* numbering) with the 2'-OH group of the mRNA codon bound with the tRNA anticodon at the P site [75]. Remarkably, this suggestion obtained an excellent confirmation from the recently deposited model of the discussed above yeast 48S PIC (PDB accession code 3J81) [58]. Careful inspection of this model shows that the above-mentioned rRNA phosphate is located at a distance <4 Å from 2'-OH group of the adenine of the AUG codon base paired with Met-tRNA_i_ at the P site.

**Figure 7:**
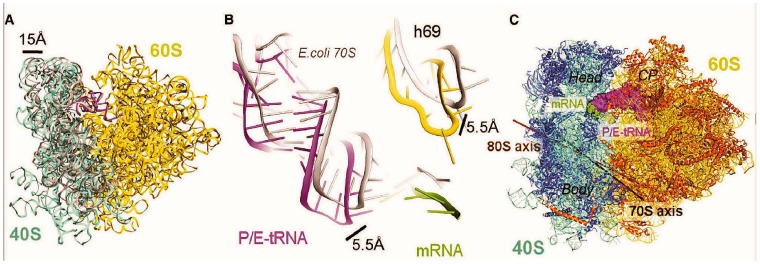
Differences in the conformations of rotated yeast 80S and bacterial 70S ribosomes in the complex with P/E-site tRNA [18]. (**A**) The tilt of the small 40S subunit relative to the large 60S subunit resulting in the shift of the head of the small subunit away from the core of the 60S subunit by up to 15 Å. 18S and 25S rRNAs in the rotated yeast ribosome are shown in blue and yellow, respectively; *E. coli* 16S rRNA of the 30S subunit in the analogous bacterial complex is shown in gray; (**B**) Differences in positions of the tRNA, mRNA, and helix 69 between the rotated yeast and *E. coli* ribosomes. Components of the bacterial complex are presented in gray; (**C**) Intersubunit rotation axes for yeast (brown) and *E. coli* (black) ribosomes (CP, central protuberance of the large subunit).

Recent 6 Å resolution cryo-EM study of the yeast complex with single deacylated tRNA at the P site [18] has confirmed the discussed biochemical data and, besides, revealed a number of details of tRNA-ribosome interactions involving tRNA parts other than the CCA-terminus. It was found that the ribosome in the complex of this type adopts a single rotated conformation with tRNA position very similar to that in the rabbit PRE complexes [14]. Comparison between 70S and 80S complexes containing single deacylated tRNA at the P site showed that positioning of tRNA in these complexes is significantly dissimilar [18] (Figure 7). Eukaryote-specific peculiarities of the complex concerned the configurations of the small subunit, tRNA and 25S rRNA helix H69, which is a component of an intersubunit bridge B2a close to the 40S subunit decoding site. In 80S complexes, due to the tilted conformation of the 40S subunit, the ASL of P/E-site tRNA and H69 are shifted together with the small subunit closer to the E site as compared to those in 70S complexes [35].

## 5. Conclusions

Recent progress in studying structures of eukaryotic translational complexes with high resolution cryo-EM, and in particular cases, with X-ray crystallography and site-directed cross-linking has provided considerable data on interaction of tRNA with ribosomes at various steps of translation. It is now a well-documented fact that tRNA passes through various classical and hybrid binding states in the course of initiation and elongation of translation, and the variety of tRNA binding states is provided by conformational flexibility of the unique l-shaped structure of tRNA and of the ribosomal subunits. The ability of subunits to change their conformation and mutual orientation as the result of rearrangements of various types (ratchet-like movements, swivel-like rotation of the small subunit head, and rolling of the small subunit) is coupled with ability of the ribosomes to bind tRNAs in hybrid states. The majority of tRNA hybrid states (P/I, P/E, A/P and A/T) seem to be nearly identical in lower and higher eukaryotes and these states have features similar in bacterial and eukaryotic ribosomes. On the other hand, both hybrid and classical states have pronounced distinctions in ribosomes from different domains of life, and these distinctions are caused mainly by dissimilar rotational preferences of bacterial and eukaryotic ribosomes. In addition, the unusual hybrid P/pa state, never observed with prokaryotic ribosomes, has been revealed with the eukaryotic 80S initiation complex assembled on HCV IRES, suggesting that this state can take place in the course of canonical translation initiation too. In accordance with all stated above, the sets of ribosomal components interacting with tRNA at each tRNA binding site could be divided into conserved (which, in general, somewhat prevail), and eukaryote-specific. However, up to now exact functional roles of eukaryote-specific tRNA-ribosome interactions remain largely unknown and their revelation is a task for further investigations.
